# RHO-1 and the Rho GEF RHGF-1 interact with UNC-6/Netrin signaling to regulate growth cone protrusion and microtubule organization in *Caenorhabditis elegans*

**DOI:** 10.1371/journal.pgen.1007960

**Published:** 2019-06-24

**Authors:** Mahekta R. Gujar, Aubrie M. Stricker, Erik A. Lundquist

**Affiliations:** Department of Molecular Biosciences, Program in Molecular, Cellular, and Developmental Biology, University of Kansas, Lawrence, KS, United States of America; University of California San Diego, UNITED STATES

## Abstract

UNC-6/Netrin is a conserved axon guidance cue that directs growth cone migrations in the dorsal-ventral axis of *C*. *elegans* and in the vertebrate spinal cord. UNC-6/Netrin is expressed in ventral cells, and growth cones migrate ventrally toward or dorsally away from UNC-6/Netrin. Recent studies of growth cone behavior during outgrowth *in vivo* in *C*. *elegans* have led to a polarity/protrusion model in directed growth cone migration away from UNC-6/Netrin. In this model, UNC-6/Netrin first polarizes the growth cone via the UNC-5 receptor, leading to dorsally biased protrusion and F-actin accumulation. UNC-6/Netrin then regulates protrusion based on this polarity. The receptor UNC-40/DCC drives protrusion dorsally, away from the UNC-6/Netrin source, and the UNC-5 receptor inhibits protrusion ventrally, near the UNC-6/Netrin source, resulting in dorsal migration. UNC-5 inhibits protrusion in part by excluding microtubules from the growth cone, which are pro-protrusive. Here we report that the RHO-1/RhoA GTPase and its activator GEF RHGF-1 inhibit growth cone protrusion and MT accumulation in growth cones, similar to UNC-5. However, growth cone polarity of protrusion and F-actin were unaffected by RHO-1 and RHGF-1. Thus, RHO-1 signaling acts specifically as a negative regulator of protrusion and MT accumulation, and not polarity. Genetic interactions are consistent with RHO-1 and RHGF-1 acting with UNC-5, as well as with a parallel pathway, to regulate protrusion. The cytoskeletal interacting molecule UNC-33/CRMP was required for RHO-1 activity to inhibit MT accumulation, suggesting that UNC-33/CRMP might act downstream of RHO-1. In sum, these studies describe a new role of RHO-1 and RHGF-1 in regulation of growth cone protrusion by UNC-6/Netrin.

## Author summary

Neural circuits are formed by precise connections between axons. During axon formation, the growth cone leads the axon to its proper target in a process called axon guidance. Growth cone outgrowth involves asymmetric protrusion driven by extracellular cues that stimulate and inhibit protrusion. How guidance cues regulate growth cone protrusion in neural circuit formation is incompletely understood. This work shows that the signaling molecule RHO-1 acts downstream of the UNC-6/Netrin guidance cue to inhibit growth cone protrusion in part by excluding microtubules from the growth cone, which are structural elements that drive protrusion.

## Introduction

The connectivity of neuronal circuits is established through properly guided axons which form functional synaptic connections. The growing axon is guided to its target by the motile, actin-based growth cone at the tip of the growing neurite. Growth cone response to extracellular guidance cues allows the axon to extend, retract, turn and branch, regulated by the reorganization and dynamics of the actin and microtubule cytoskeletons of the growth cone [1].

In *C*. *elegans* and vertebrates, the conserved laminin-like UNC-6/Netrin guidance cue and its receptors UNC-40/DCC and UNC-5 direct dorsal-ventral axon outgrowth [2–10]. UNC-6 is secreted by cells in the ventral nerve cord [11], and growth cones grow toward UNC-6/Netrin (i.e. ventral migration; attraction) and away from UNC-6/Netrin (i.e. dorsal migration; repulsion). The prevailing model of UNC-6/Netrin-mediated axon guidance involves a ventral-to-dorsal chemotactic gradient of the molecule, which growth cones interpret by migrating up or down the gradient using the “attractive” receptor UNC-40/DCC or the “repulsive” receptor UNC-5, respectively [12, 13]. However, this model has recently been challenged by studies in mouse spinal cord showing that floorplate Netrin is dispensable for commissural axon guidance, and that ventricular expression is important, possibly in a close-range, haptotactic event [14–17].

Experiments leading to the statistically-oriented asymmetric localization (SOAL) model in neurons with growth cones that grow ventrally toward UNC-6 were among the first studies to show that UNC-6/Netrin gradients were not required to explain directed outgrowth [18–20]. In the HSN neuron, which extends an axon ventrally, UNC-6/Netrin controls the biased ventral accumulation of the UNC-40 receptor in the HSN cell body, and UNC-5 acts to bias UNC-40/DCC ventrally, resulting in probabilistic bias of protrusion to the ventral surface [18–20]. Our previous work with the VD growth cones that migrate dorsally (repelled) suggests that UNC-6/Netrin first polarizes protrusion and F-actin to the dorsal side of the growth cone via the UNC-5 receptor, and then regulates protrusion based on this polarity (the polarity/protrusion model). UNC-5 inhibits protrusion ventrally, close to the UNC-6/Netrin source, and UNC-40 stimulates protrusion dorsally, away from the UNC-6/Netrin source, resulting in directed dorsal growth away from UNC-6/Netrin [21–23]. That polarity and protrusion are separable events was suggested previously in HSN by missense mutations in UNC-6 and UNC-40 that uncouple their roles in polarity and migration [24]. Neither the SOAL model in ventrally-growing axons or the polarity/protrusion model in dorsally growing axons rely on chemotactic gradients and instead involve growth cone asymmetries coupled with regulation of protrusive growth by these asymmetries. Chemotactic gradient models imply a tight coupling of growth cone polarity and protrusion (i.e. different concentrations of UNC-6/Netrin lead to different protrusive activities across the growth cone). While the SOAL model is based on asymmetry of axon initiation in the HSN cell body, and the polarity/protrusion model is based on analyzing growth cones during outgrowth, the idea of separability of polarity and protrusion in directed migration is similar in both models. Also similar in both models is that the UNC-5 receptor, considered the “repulsive” receptor in classical gradient models, acts in both growth toward and away from UNC-6/Netrin.

UNC-40/DCC drives growth cone lamellipodial and filopodial protrusion via the small GTPases CDC-42, CED-10/Rac, and MIG-2/RhoG, the Rac-specific guanine nucleotide exchange factor (GEF) TIAM-1, and actin cytoskeletal regulators Arp2/3, UNC-34/Enabled and UNC-115/abLIM [25–29]. UNC-5 inhibits growth cone protrusion via the Rac GEF UNC-73/trio, CED-10/Rac and MIG-2/RhoG (also used to drive protrusion), the FMO flavin monooxygenases which might act via actin, and the actin and MT-interacting proteins UNC-33/CRMP and UNC-44/Ankyrin [22, 23, 30]. UNC-5 also restricts the accumulation of microtubule + ends in VD growth cones which have pro-protrusive effects [21]. Thus, in *unc-5* mutants, VD growth cones are larger and more protrusive, display unpolarized protrusion including ventral protrusions, display unpolarized F-actin around the periphery of the growth cone, and have increased accumulation of MT+ ends [21, 22]. This unregulated protrusion results in unfocused growth cones that fail to migrate dorsally away from UNC-6/Netrin, causing the severe VD axon guidance defects seen in *unc-5* mutants.

The Rho-family GTPases CED-10/Rac, MIG-2/RhoG, and CDC-42 control neuronal protrusion [23, 26, 28, 31]. Here we dissect the role of RHO-1, the single RhoA molecule encoded in the *C*. *elegans* genome, in regulation of VD growth cone polarity and protrusion. *rho-1* RNAi results in early embryonic arrest, with a failure in cytokinesis and severe morphological defects [32–35]. We used cell-specific expression of constitutively-active RHO-1(G14V) and dominant-negative RHO-1(T19N), and cell-specific RNAi of *rho-1* and found that RHO-1 inhibited growth cone protrusion and MT+ end accumulation. RHO-1 did not, however, affect polarity of protrusion or F-actin, demonstrating that growth cone polarity can be separated from growth cone protrusion. We also found that the RHO-1 activator RHGF-1, a RHO-1 GTP exchange factor of the LARG family [36, 37], was required to inhibit protrusion and MT+ end accumulation similar to RHO-1. Genetic interactions with UNC-5 signaling and UNC-33/CRMP suggest that RHGF-1 and RHO-1 might act downstream of UNC-5 and in parallel to other regulators of protrusion and MT+ end accumulation. These studies also revealed that RHO-1 requires UNC-33/CRMP to prevent MT+ end accumulation. In sum, results reported here show that RHGF-1 and RHO-1 are key inhibitors of growth cone protrusion and MT+ end accumulation and act with UNC-5 in protrusion, but not growth cone polarity.

## Results

### RHO-1 regulates growth cone protrusion but not polarity

**Fig 1:**
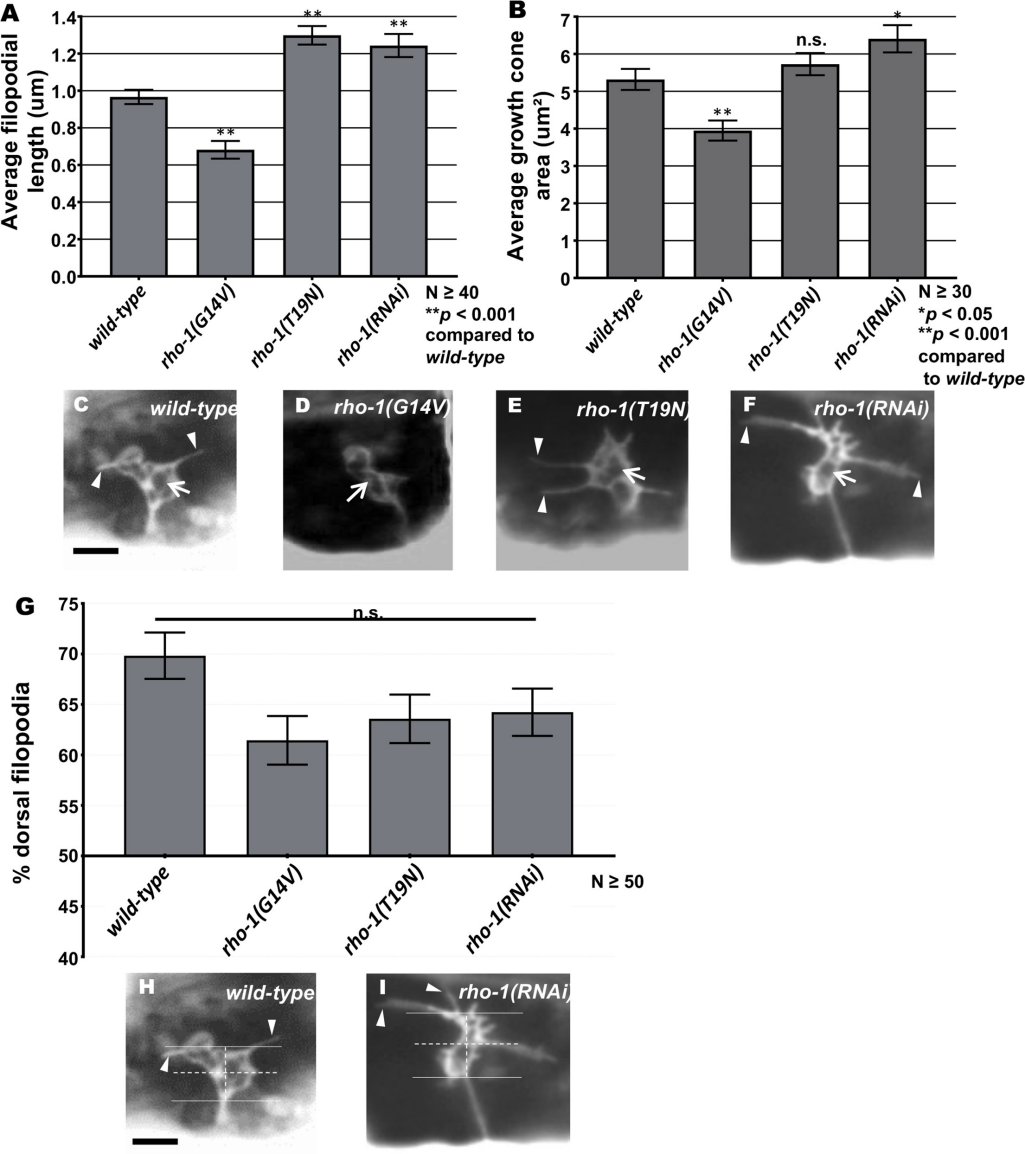
VD growth cone protrusion and polarity in *rho-1* mutants. (A-B) Quantification of VD growth cone filopodial length and growth cone area in wild-type and *rho-1* mutant animals (See Materials and Methods). (A) Average filopodial length, in μm. (B) Growth cone area in μm^2^. Error bars represent 2x standard error of the mean; asterisks indicate the significant difference between wild-type and the mutant phenotype (*p < 0.05, **p < 0.001) determined by Analysis of Variance (ANOVA). n.s., not significant. (C-E) Fluorescence micrographs of VD growth cones with *Punc-25*::*gfp* expression (*juIs76*); (C) A wild-type VD growth cone. (D) *rho-1(G14V)* showing small and inhibited VD growth cone phenotype (E) *rho-1(T19N)* and (F) *rho-1(RNAi)* growth cones showing increased filopodial protrusion in the form of longer filopodia. Arrows point to the growth cone and arrow heads indicate representative filopodia. (G) A graph showing the percent of dorsally-directed filopodial protrusions in VD growth cones of different genotypes (see Materials and Methods). (H-I) VD growth cones with *Punc-25*::*gfp* expression (*juIs76*). The solid horizontal lines indicate the dorsal and ventral extent of the growth cone body, and the hatched lines indicate the average center of the growth cone. Protrusions above the hatched horizontal line are considered dorsal, and those below ventral. Scale bars represent 5μm.

RHO-1 is the single RhoA homolog in *C*. *elegans*. Loss of *rho-1* leads to embryonic lethality, with a failure in cytokinesis [38], and perturbation of RHO-1 signaling in adults results in dysfunction in numerous neuronal and non-neuronal functions leading to death [39]. To understand the role of RHO-1 in VD growth cone morphology, we constructed constitutively-active G14V and dominant-negative T19N versions of RHO-1, and expressed them in the VD/DD neurons using the *unc-25* promoter. Constitutively-active *rho-1(G14V)* expression significantly reduced the VD growth cone area and shortened filopodial protrusions as compared to wild-type (Fig 1A, 1B and 1D). In contrast, dominant-negative *rho-1(T19N)* expression displayed significantly longer filopodial protrusions as compared to wild-type VD growth cones (Fig 1A, 1B and 1E). Growth cone area was increased, but not significantly so. These results indicate that RHO-1 activity inhibits growth cone protrusion.

We used a transgenic RNAi approach to knock down *rho-1* in the VD/DD motor neurons as previously described (see Materials and Methods) [40, 41]. Plasmids were generated to drive expression of sense and antisense RNA fragments complementary to the *rho-1* under the control of the *unc-25* promoter. Animals were made transgenic with a mix of the sense and antisense plasmids, and the resulting transgenes were used in analysis. The average length of filopodial protrusions and growth cone area were significantly increased in *rho-1(RNAi)* (Fig 1A, 1B and 1F). These data suggest that RHO-1 normally inhibits VD growth cone protrusion. The polarity of filopodial protrusions was not affected by *rho-1(DN)* or *rho-1(RNAi)*, as protrusions still displayed a dorsal bias similar wild-type (Fig 1G–1I). Thus, despite showing increased protrusion, the polarity of growth cone protrusion was not affected by *rho-1*.

*rho-1(G14V)*, *rho-1(T19N)*, and *rho-1(RNAi* each resulted in low-penetrance but significant VD/DD axon guidance defects (Table 1), including wandering, branching, and failing to reach the dorsal nerve cord. This suggests that the effects of RHO-1 on the growth cone result in axon guidance defects.

**Table 1:** VD/DD axon guidance defects.

Genotype (n = 100 animals; 1600 axons)	% defective VD/DD axon guidance
*wild-type*	1.5
*rho-1(G14V)*	5.1*
*rho-1(T19N)*	5.3*
*rho-1(RNAi)*	9.8*
*rhgf-1(gk217)*	6.6*
*rhgf-1(ok880)*	9.0*
*rhgf-1(gk292502)*	6.7*
*rhfg-1(gk217); rho-1(T19N)*	14.7**
*rhfg-1(gk217); rho-1(RNAi)*	13.5
*rhfg-1(ok880); rho-1(T19N)*	14.2
*rhfg-1(ok880); rho-1(RNAi)*	11.4
	% VD/DD failure to cross lateral midline
*wild-type*	0.0
*rho-1(T19N)*	0.0
*rho-1(RNAi)*	0.0
*unc-5(e152)*	12.3
*unc-5(e152); rho-1(T19N)*	39.9***
*unc-5(e152); rho-1(RNAi)*	44.4***

* *p* < 0.0001 compared to *wild-type*.

** p = 0.006 compared to the additive effect of *rhgf-1(gk217)* and

*rho-1(T19N)*.

*** p < 0.0001 compared to *unc-5(e152)* alone.

### RHO-1 is required to limit EBP-2::GFP puncta accumulation in VD growth cones

**Fig 2:**
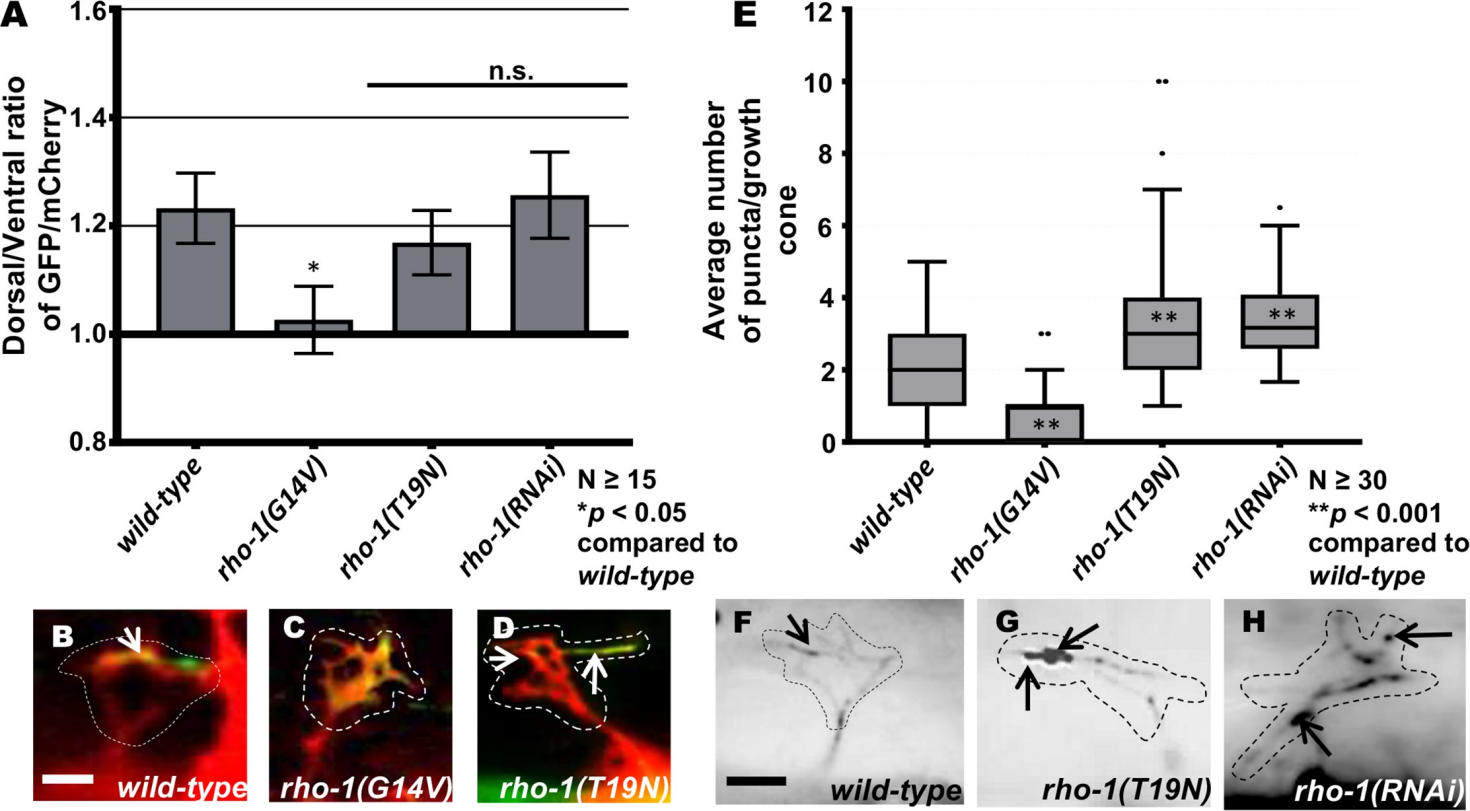
VD growth cone F-actin polarity and EBP-2::GFP accumulation in *rho-1* mutants. (A) The average dorsal/ventral ratio of GFP/mCherry from multiple growth cones in wild-type and mutant animals expressing VAB-10ABD::GFP and mCherry (a volumetric marker) as described previously [22] (see Materials and Methods) Error bars represent 2x standard error of the mean. Asterisks (*) indicate the significant difference between wild-type and the mutant phenotype (*p < 0.05) determined by ANOVA. (B-D) Representative images of VD growth cones with cytoplasmic mCherry in red (a volumetric marker) and VAB-10ABD::GFP in green. Areas of overlap are yellow (arrows). Dashed lines indicate the growth cone periphery. Dorsal is up and anterior is left. Scale bar: 5 μm. (B) A wild-type VD growth cone, (C) *rho-1(G14V)* showing an inhibited growth cone with F-actin accumulation all along the growth cone and (D) *rho-1(T19N)* VD growth cones with VAB-10ABD::GFP expression in the dorsal leading edge of the growth cone. (E) Box-and-whiskers plot of the number of EBP-2::GFP puncta in the growth cones of different genotypes (≥25 growth cones for each genotype). The grey boxes represent the upper and lower quartiles, and error bars represent the upper and lower extreme values. Dots represent outliers. Asterisks (*) indicate the significant difference between wild-type and the mutant phenotype (**p < 0.001) determined by ANOVA. n.s., not significant. (F-H) Fluorescence micrographs of EBP-2 distribution in the VD growth cones; (F) A wild-type VD growth cone and (G) *rho-1(T19N)* and (H) *rho-1(RNAi)* growth cones showing increased puncta in the growth cone and filopodial protrusions. Arrows indicate representative EBP-2::GFP puncta. Dashed lines indicate the growth cone perimeter. Dorsal is up and anterior is left. Scale bar: 5μm.

Previous studies indicate that in VD growth cones, F-actin accumulates at the dorsal, protrusive edge of the growth cone and acts as a polarity mark to specify protrusion in this region (Fig 2A and 2B) [21, 22]. Furthermore, microtubule + ends are present in the growth cone and have a pro-protrusive role [21]. In wild-type, MT+ ends are rare in VD growth cones (~2 per growth cone) (Fig 2E and 2F) [21], and protrusion is tightly regulated and localized to the dorsal leading edge at the site of F-actin accumulation Fig 2) [21].

VD growth cone F-actin was monitored using the VAB-10ABD::GFP reporter, and MT+ ends were monitored using EBP-2::GFP as described previously [21, 22]. Dominant-negative *rho-1(T19N)* and *rho-1(RNAi)* had no effect on dorsally-polarized F-actin accumulation (Fig 2A and 2D), consistent with no effects on growth cone polarity of protrusion (Fig 1). However, growth cone EBP-2::GFP puncta number were significantly increased by dominant-negative *rho-1(T19N)* and *rho-1(RNAi)* (Fig 2E, 2G and 2H), consistent with increased protrusion in these backgrounds.

Constitutively-active *rho-1(G14V)* resulted in fewer EBP-2::GFP puncta, consistent with reduced growth cone protrusion (Fig 2E). F-actin polarity was also abolished, with distribution along the periphery of the entire growth cone (Fig 2A and 2C). Possibly, constitutive activation reveals a role of RHO-1 in F-actin polarity that is not affected in reduction of function treatments. However, a similar effect on F-actin was observed with constitutively-active Rac GTPases MIG-2 and CED-10 [21]. Possibly, this effect on F-actin is a consequence of small growth cones with severely-restricted protrusion, and not a direct role in F-actin organization. In sum, these results suggest that RHO-1 normally restricts growth cone protrusion by preventing accumulation of growth cone MT+ ends.

### The RhoGEF RHGF-1 acts with RHO-1 to inhibit growth cone filopodial protrusion and MT+ end accumulation

**Fig 3:**
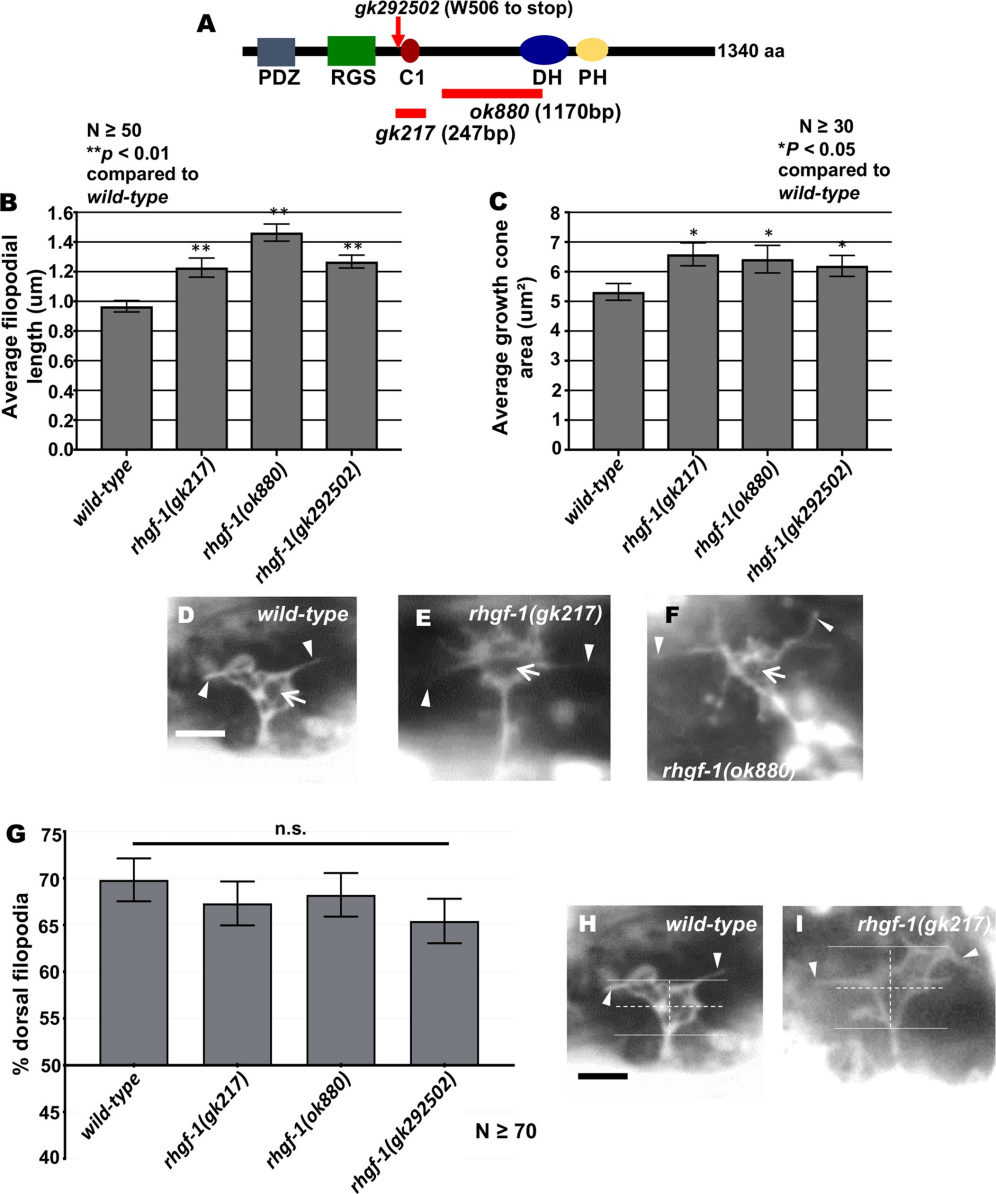
Growth cone protrusion and polarity in *rhgf-1* loss-of-function. (A) A schematic diagram of the predicted 1,340-amino acid residue RHGF-1 molecule. PDZ = PDZ domain, RGS = Regulator of G protein signaling domain, C1 = Ester/diacylglycerol binding domain, DH = Dbl homology domain, PH = Plekstrin homology domain. Extent of deletions of *ok880* and *gk217* are indicated the red lines. The red arrow points to the premature stop site in *gk292502*. (B-C) Quantification of VD growth cone filopodial length and growth cone area as described in Fig 1. *p < 0.05 and **p < 0.001, determined by ANOVA. n.s., not significant. (D-F) Fluorescence micrographs of VD growth cones (*juIs76[Punc-25*::*gfp]*). Arrows point to the growth cone and arrow heads indicate representative filopodia. Scale bar: 5μm. (G) A graph showing the percent of dorsally-directed filopodial protrusions in VD growth cones of different genotypes as described in Fig 1. (H-I) Growth cone polarity of protrusion as described in Fig 1.

RHGF-1 is a PDZ RhoGEF with PDZ, RGS, C1, DH, and PH domains (Fig 3A). RHGF-1 is a RHO-1-specific GEF and acts with RHO-1 in neurotransmitter release and axonal regeneration [36, 37, 42–44]. *rhgf-1(ok880)* is a 1170bp in frame deletion which removes a large part of the DH domain and is predicted to have no RhoGEF activity [24], *rhgf-1(gk217)* is a 247bp in frame deletion which removes the C1 domain, and *rhgf-1(gk292502)* produces a premature stop just before the C1 domain (Fig 3A). *rhgf-1* mutants each displayed increased growth cone area and longer filopodial protrusions compared to wild-type (Fig 3B–3F). The dorsally-biased polarity of growth cone protrusion was not significantly affected by *rhgf-1* mutation (Fig 3G–3I). These data indicate that RHGF-1 is normally required to limit the extent of growth cone protrusion, but does not regulate growth cone polarity, similar to *rho-1*. *rhgf-1* mutants displayed low-penetrance but significant VD/DD axon guidance defects (Table 1), suggesting that the effects of *rhgf-1* on the growth cone has ramifications on axon guidance.

**Fig 4:**
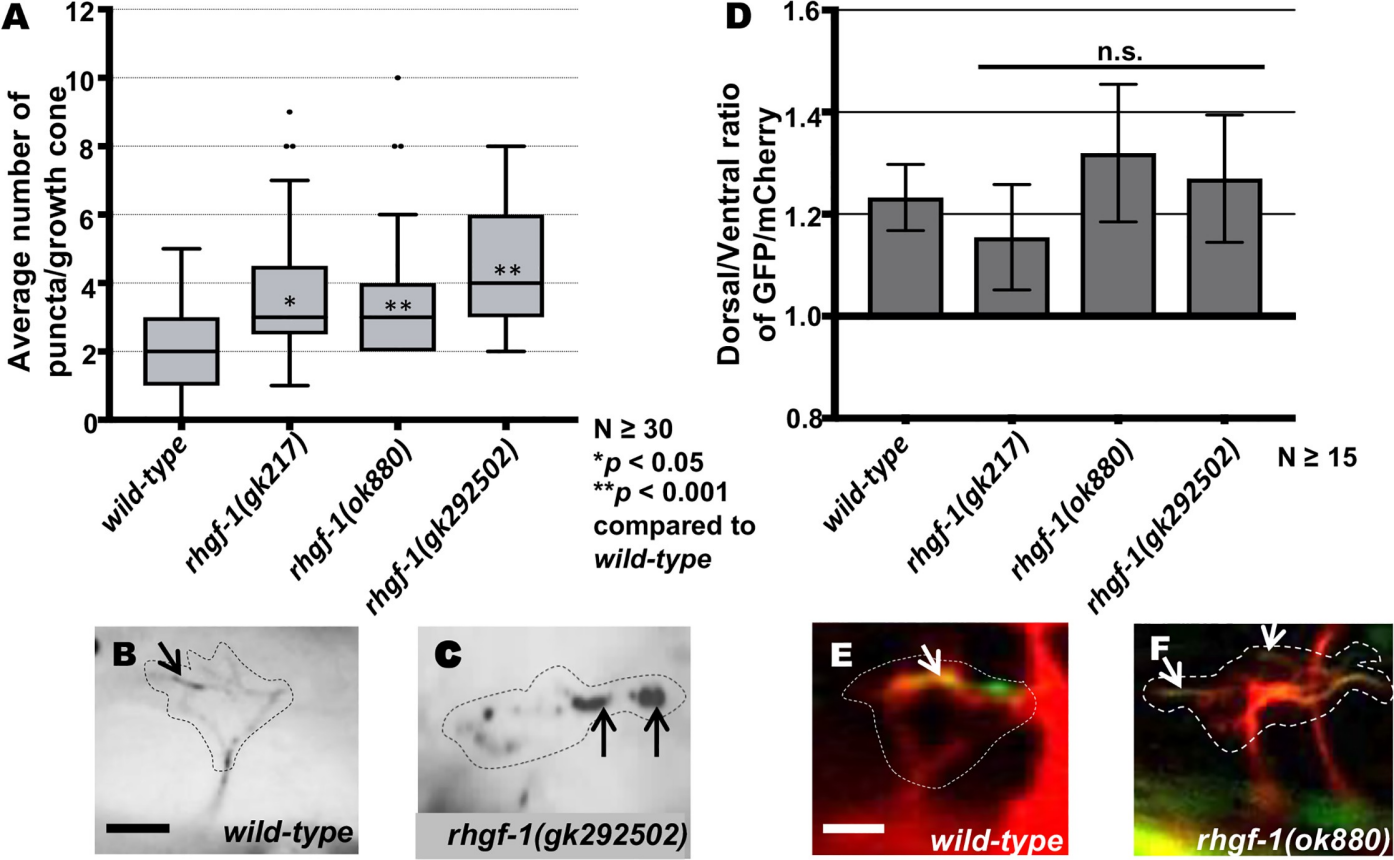
EBP-2::GFP accumulation and F-actin polarization in *rhgf-1* mutants. (A) Quantification of the number of EBP-2::GFP puncta in wild-type and *rhgf-1* mutant growth cones as described in Fig 2E. Asterisks (*) indicate the significant difference between wild-type and the mutant phenotype (*p < 0.05,**p < 0.001) determined by ANOVA. n.s., not significant. (B-C) Fluorescence micrographs of EBP-2 distribution in the VD growth cones; (B) A wild-type VD growth cone (C) *rhgf-1(gk292502) g*rowth cones showing increased puncta in the growth cone and filopodial protrusions. Arrows indicate representative EBP-2::GFP puncta. Dashed lines indicate the growth cone perimeter. Dorsal is up and anterior is left. Scale bar: 5μm. (D) The average dorsal-to-ventral ratio of VAB-10ABD::GFP/mCherry from multiple growth cones in wild-type and mutant animals as described in Fig 2A. Error bars represent 2x standard error of the mean; n.s., not significant. (E-F) Representative images of VD growth cones with cytoplasmic mCherry in red (a volumetric marker) and the VAB-10ABD::GFP in green. Areas of overlap are yellow (arrows). Dashed lines indicate the growth cone periphery. Dorsal is up and anterior is left. Scale bar: 5 μm. (E) A wild-type growth cone and (F) *rhgf-1(ok880)* growth cones with VAB-10ABD::GFP expression in the dorsal leading edge of the growth cone.

The *Drosophila* RHGF-1 homolog DRhoGEF2 is a key regulator of morphogenesis and associates with the tips of growing MTs and exhibits plus end tracking [45]. In *C*. *elegans*, RHGF-1 associates with MTs and initiates an axon regeneration pathway [37]. *rhgf-1* mutant VD growth cones displayed significantly increased numbers of EBP-2::GFP puncta (Fig 4A–4C), but caused no significant defects in F-actin organization, similar to *rho-1* knockdown (Fig 4D–4F). These results indicate that RHGF-1 might act with RHO-1 to inhibit growth cone protrusion by excluding MT+ ends from entering the growth cone periphery.

**Fig 5:**
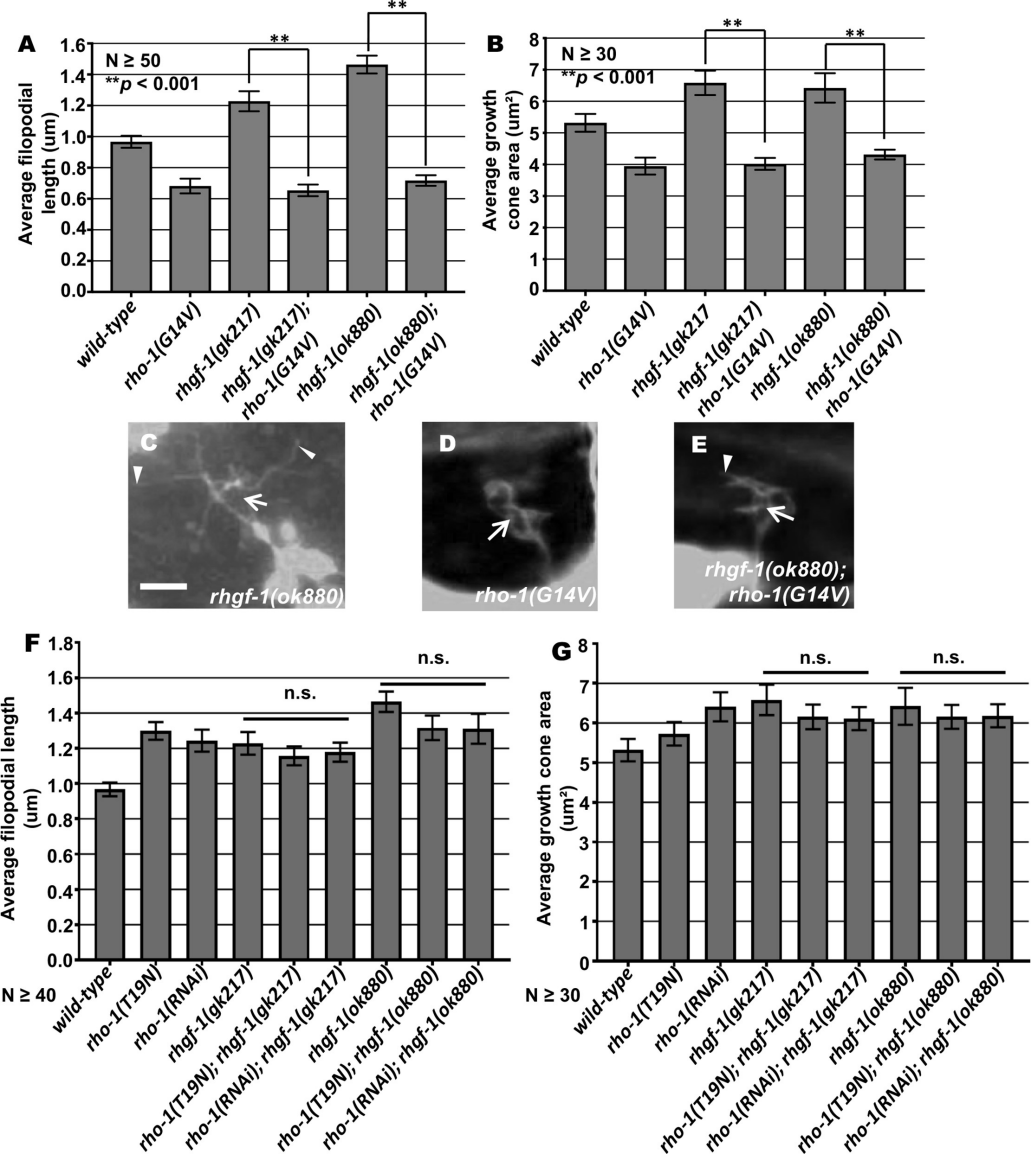
Genetic interactions of *rhgf-1* and *rho-1* in growth cone protrusion. (A-B) Quantification of VD growth cone filopodial length and growth cone area in single and double mutant animals as described in Fig 1. (A) Average filopodial length, in μm. (B) Growth cone area in μm^2^. Error bars represent 2x standard error of the mean; asterisks indicate the significant difference between *rhgf-1* single mutants and the double mutant phenotype (**p < 0.001) determined by ANOVA. (C-E) Fluorescence micrographs of VD growth cones as described in Fig 1. Arrows point to the growth cone and arrow heads indicate representative filopodia. Scale bar: 5μm. (F-G) Quantification of growth cone filopodial length and growth cone area as described in Fig 1. While double mutants are significantly different than each single alone, the effects are additive.

**Fig 6:**
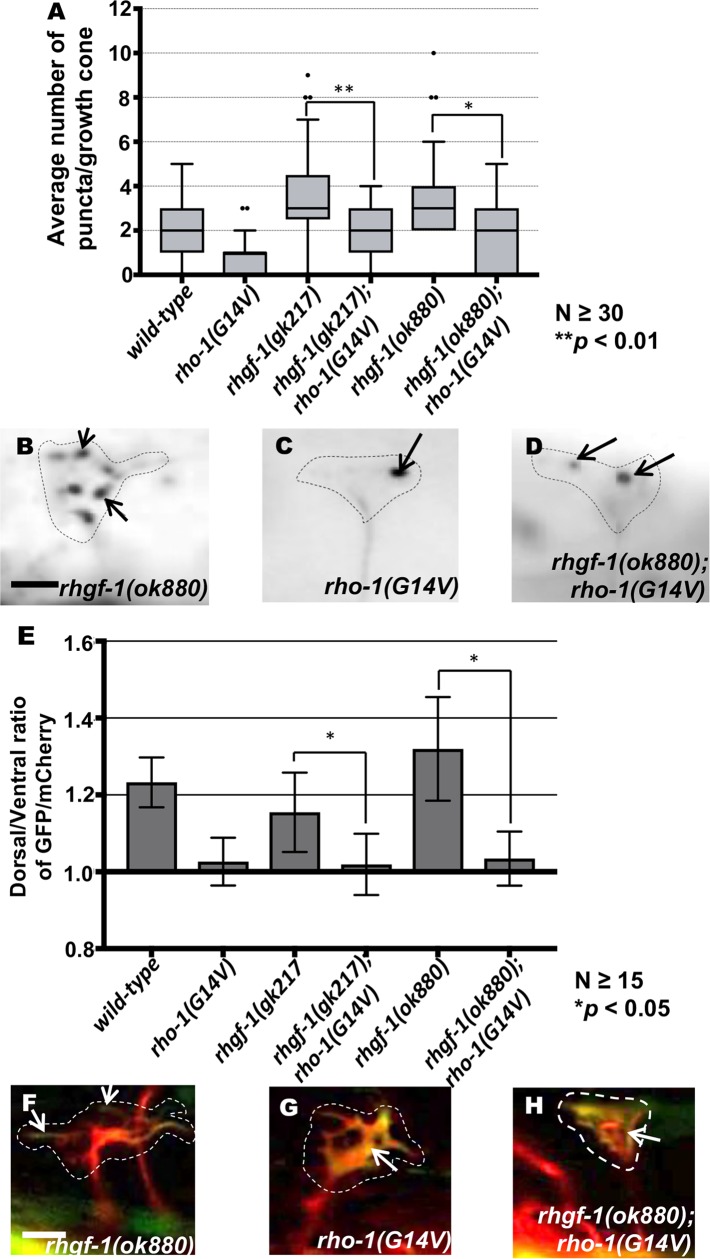
Genetic interactions of *rhgf-1* and *rho-1* in EBP-2::GFP accumulation and F-actin polarization. **(A)** Quantification of the number of EBP-2::GFP puncta in wild-type and mutant animals as described in Fig 2E. Asterisks (*) indicate the significant difference between *rhgf-1* single mutants and the double mutant phenotype (**p < 0.01) determined by ANOVA. (B-D) Fluorescence micrographs of EBP-2 distribution in the VD growth cones; (B) *rhgf-1(ok880)* growth cone (C) *rho-1(G14V)* and (D) *rhgf-1(ok880); rho-1(G14V) g*rowth cones with decreased *ebp-2* puncta. Arrows indicate representative EBP-2::GFP puncta. Dashed lines indicate the growth cone periphery. Dorsal is up and anterior is left. Scale bar: 5 μm. (E) The average dorsal-to-ventral ratio of GFP/mCherry from multiple growth cones in single and double mutant animals as described in Fig 2A. (F-H) Representative images of VD growth cones with cytoplasmic mCherry in red (a volumetric marker) and the VAB-10ABD::GFP in green. Areas of overlap are yellow (arrows). Error bars represent 2x standard error of the mean. Asterisks (*) indicate the significant difference between single and double mutant phenotype (*p < 0.05) determined by ANOVA. Dashed lines indicate the growth cone periphery. Dorsal is up and anterior is left. Scale bar: 5 μm.

The results above indicate that the VD growth cones of activated *rho-1(G14V)* displayed reduced protrusion, and that those of *rhgf-1* loss of function were overly-protrusive. The VD growth cones of activated *rho-1(G14V)* double mutants with *rhgf-1* loss of function resembled the small, inhibited growth cones of *rho-1(G14V)* alone (Fig 5A–5E), with a significant reduction in filopodial length and growth cone area as compared to *wild-type* and *rhgf-1* mutants alone (Fig 5A–5E). Similarly, double mutants of *rhgf-1* and *rho-1(G14V)* showed a significant decrease in the average number of EBP-2 puncta in the growth cone similar to *rho-1(G14V)* alone (Fig 6A–6D). VAB-10ABD::GFP distribution in these double mutant growth cones also resembled activated *rho-1(G14V)* with F-actin distributed randomly all across the growth cone (Fig 6E–6H). That activated RHO-1(G14V) was epistatic to *rhgf-1* loss of function is consistent with RHO-1 acting downstream of RHGF-1 in limiting growth protrusion and EBP-2 accumulation in VD growth cones.

Double mutants of dominant-negative *rho-1(T19N)* and *rho-1(RNAi)* with *rhgf-1* did not result in significant enhancement of growth cone protrusion compared to single mutants (Fig 5F and 5G). VD/DD axon guidance defects were also not enhanced, except in one case (Table 1). These results further support the idea that RHO-1 and RHGF-1 act in the same pathway in growth cone protrusion and axon guidance.

### Activated *myr*::*unc-40* and *myr*::*unc-5* require RHGF-1

**Fig 7:**
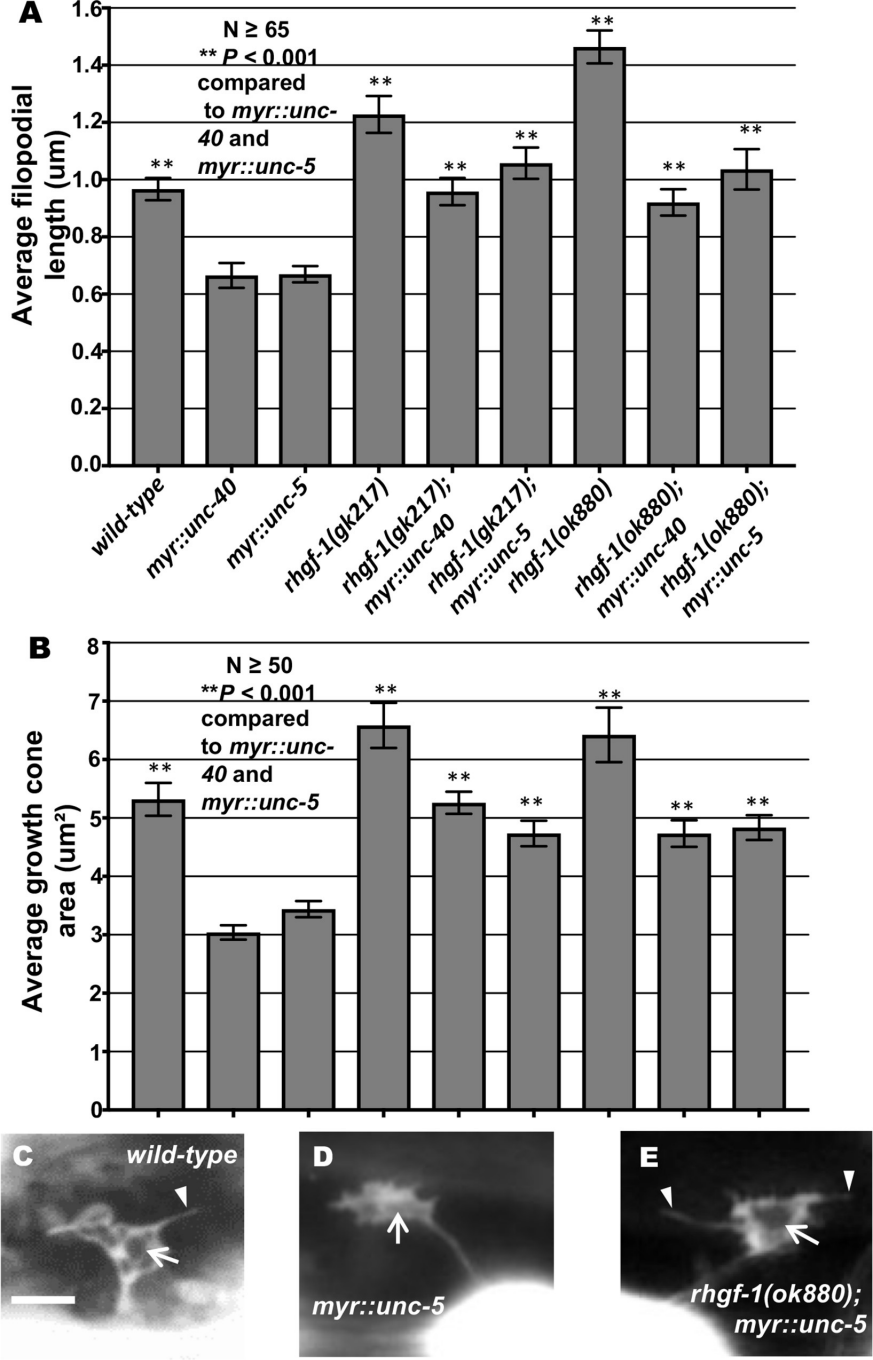
Genetic interactions of *rhgf-1* with *myr*::*unc-40* and *myr*::*unc-5* in growth cone protrusion. (A) Quantification of VD growth cone filopodial length and growth cone area in single and double mutant animals as described in Fig 1. (A) Average filopodial length, in μm. (B) Growth cone area in μm^2^. Error bars represent 2x standard error of the mean; asterisks indicate the significant difference between *myr*::*unc-40*, single and double mutants (**p < 0.001) determined by ANOVA. Genotypes on the X-axis are as listed in A. (C-E) Fluorescence micrographs of mutant VD growth cones as described in Fig 1. Arrows point to the growth cone and arrow heads indicate representative filopodia. Scale bar: 5μm.

Previous studies showed that UNC-6/Netrin signaling via the heterodimeric UNC-40/UNC-5 receptor is required for inhibition of growth cone protrusion in UNC-6/Netrin repulsive axon guidance [22, 23]. Constitutive activation of UNC-40 and UNC-5 using myristoylated versions of the cytoplasmic domains of UNC-40 and UNC-5 (*myr*::*unc-40* and *myr*::*unc-5*) in the VD neurons result in small growth cones with few or no filopodial protrusions [22, 23, 25]. Loss of *rhgf-1* significantly suppressed inhibition of filopodial protrusion and growth cone size caused by *myr*::*unc-40* and *myr*::*unc-5* (Fig 7).

**Fig 8:**
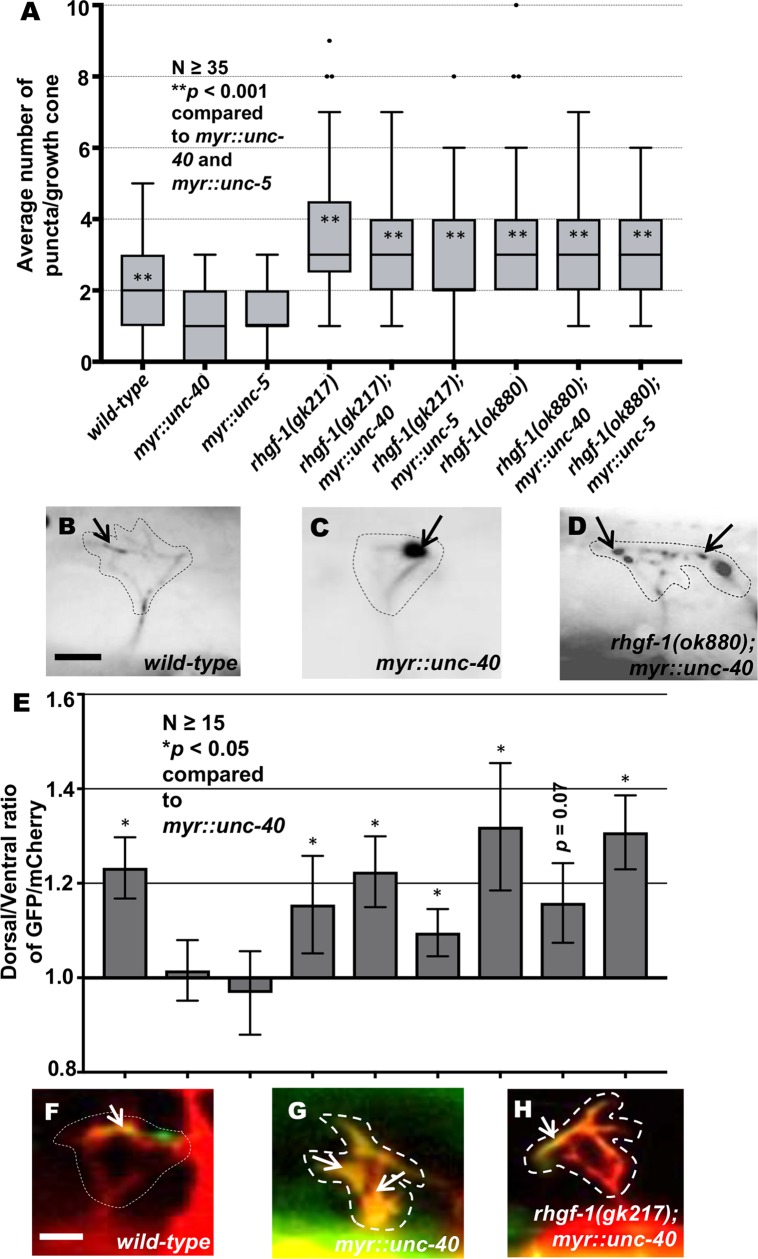
Genetic interactions of *rhgf-1* with *myr*::*unc-40* and *myr*::*unc-5* in EBP-2::GFP accumulation and F-actin polarity. (A) Quantification of the number of EBP-2::GFP puncta in wild-type and mutant animals as described in Fig 2E. Asterisks (*) indicate the significant difference between *myr*::*unc-40*, single mutants and double mutants (**p < 0.001), determined by ANOVA. (B-E) Fluorescence micrographs of EBP-2 distribution in the VD growth cones. Arrows indicate representative EBP-2::GFP puncta. Dashed lines indicate the growth cone periphery. Dorsal is up and anterior is left. Scale bar: 5 μm. (E) The average dorsal-to-ventral ratio of GFP/mCherry from multiple growth cones in wild-type, single and double mutant animals as described in Fig 2A. Error bars represent 2x standard error of the mean. Asterisks (*) indicate the significant difference between *myr*::*unc-40*, single mutants and double mutants (*p < 0.05) determined by ANOVA. Genotypes on the X-axis are as listed in A. (F-H) Representative images of VD growth cones with cytoplasmic mCherry in red (a volumetric marker) and the VAB-10ABD::GFP in green. Areas of overlap are yellow (arrows). Dashed lines indicate the growth cone periphery. Dorsal is up and anterior is left. Scale bar: 5μm.

*myr*::*unc-40* and *myr*::*unc-5* growth cones show a significant decrease in the average number of EBP-2::GFP puncta in the VD growth cones as compared to wild-type (Fig 8A–8C) [21]. Double mutants of *rhgf-1* with *myr*::*unc-40* and *myr*::*unc-5* resembled *rhgf-1* mutants alone, with significant increases in protrusion and MT+ end accumulation (Fig 8A and 8D). Similar to activated Racs and RHO-1(G14V), F-actin is distributed throughout the small growth cones in activated *myr*::*unc-5* and *myr*::*unc-40* (Fig 8E–8G). *rhgf-1* mutation restored dorsal polarity of F-actin (Fig 8E and 8H). In sum, the growth cones of *rhgf-1* double mutants with *myr*::*unc-5* and *myr*::*unc-40* displayed increased protrusion and EBP-2 puncta accumulation compared to *myr*::*unc-40* and *myr*::*unc-5*, but normal dorsal F-actin polarity. These data indicate that RHGF-1 is required for the inhibitory effects of *myr*::*unc-40* and *myr*::*unc-5* on growth cone protrusion and EBP-2::GFP puncta accumulation.

### Activated RHO-1 does not suppress *unc-5* loss of function

**Fig 9:**
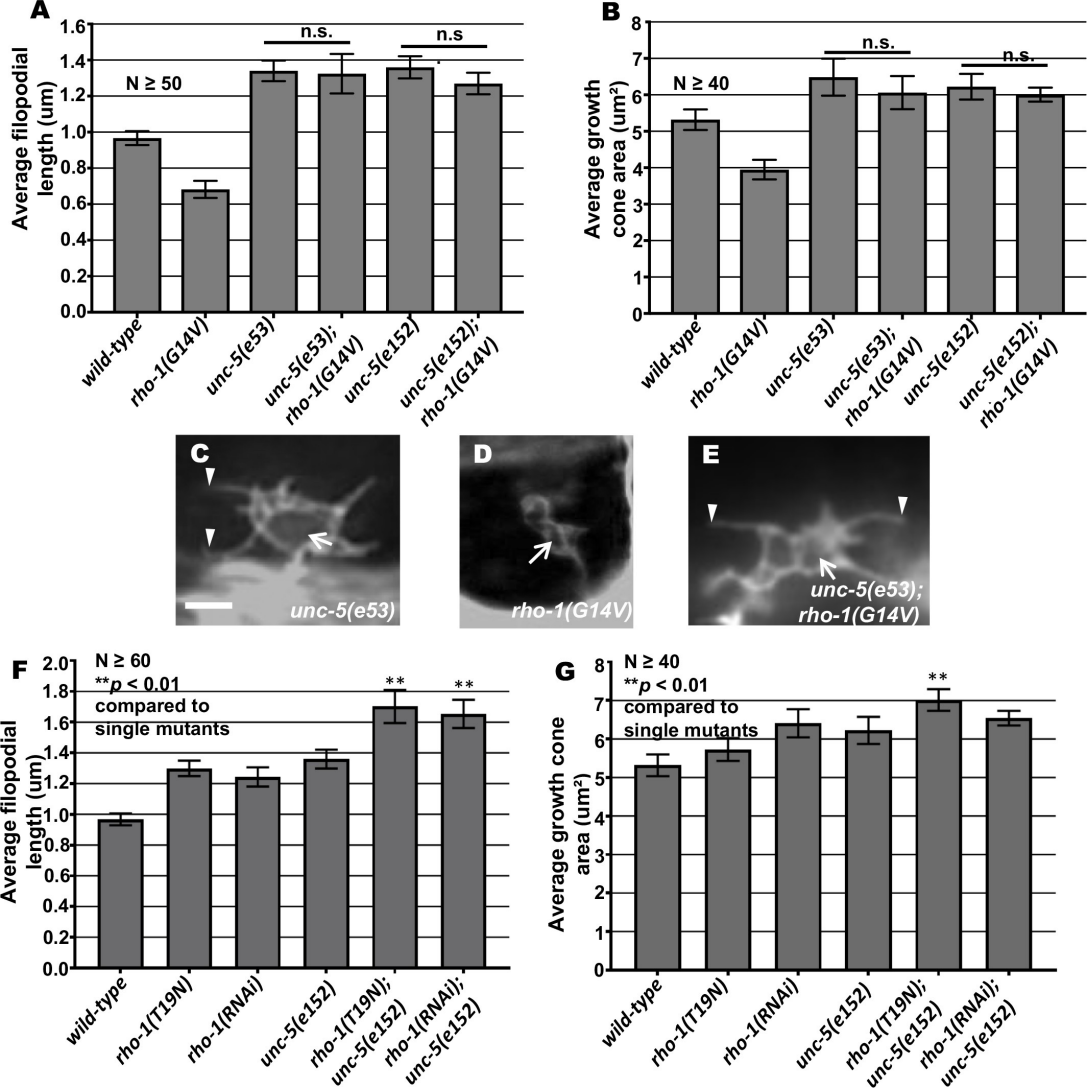
Genetic interactions of *rho-1* and *unc-5* in growth cone protrusion. (A-B) Quantification of VD growth cone filopodial length and growth cone area in single and double mutant animals as described in Fig 1. (A) Average filopodial length, in μm. (B) Growth cone area in μm^2^. Error bars represent 2x standard error of the mean; n.s., not significant determined by ANOVA. (C-E) Fluorescence micrographs of mutant VD growth cones. Arrows point to the growth cone and arrow heads indicate representative filopodia. Scale bar: 5μm. (F-G) Quantification of growth cone filopodia length and growth cone area as described in Fig 1.

**Fig 10:**
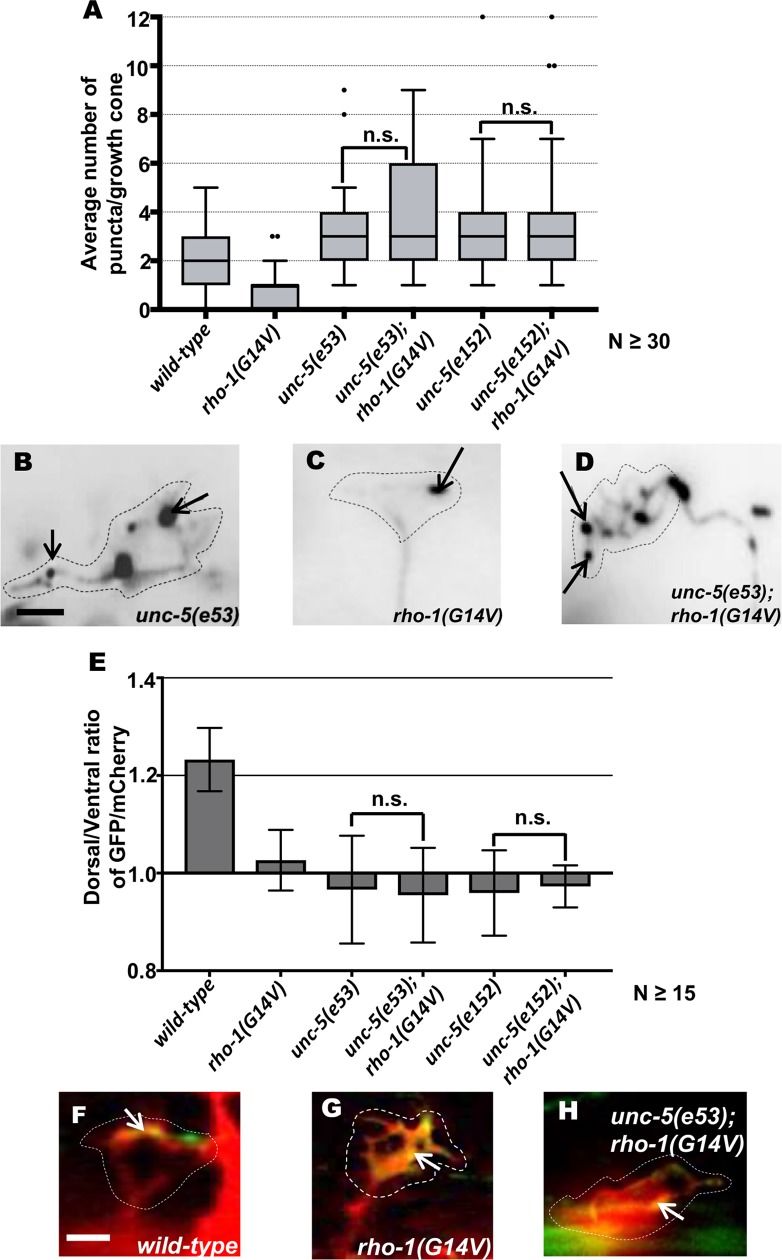
Genetic interactions of *rho-1* and *unc-5* in EBP-2::GFP accumulation and F-actin polarity. (A) Quantification of the number of EBP-2::GFP puncta in wild-type and mutant animals as described in Fig 2E. n.s., not significant, determined by two-sided *t*-test with unequal variance. (B-D) Fluorescence micrographs of EBP-2 distribution in the VD growth cones. Arrows indicate representative EBP-2::GFP puncta. Dashed lines indicate the growth cone periphery. Dorsal is up and anterior is left. Scale bar: 5 μm. (E) The average dorsal-to-ventral ratio of GFP/mCherry from multiple growth cones in wild-type, single and double mutant animals as described in Fig 2A. Error bars represent 2x standard error of the mean; n.s. indicates no significant difference between *unc-5* single mutants and double mutants determined by ANOVA. (F-H) Representative images of VD growth cones with cytoplasmic mCherry in red (a volumetric marker) and the VAB-10ABD::GFP in green. Areas of overlap are yellow (arrows). (F) wild-type growth cone, (G) *rho-1(G14V)* growth cone, (H) *unc-5(e53); rho-1(G14V)* double mutant VD growth cones with cytoplasmic mCherry and VAB-10ABD::GFP expression. Dashed lines indicate the growth cone periphery. Dorsal is up and anterior is left. Scale bar: 5 μm.

**Fig 11:**
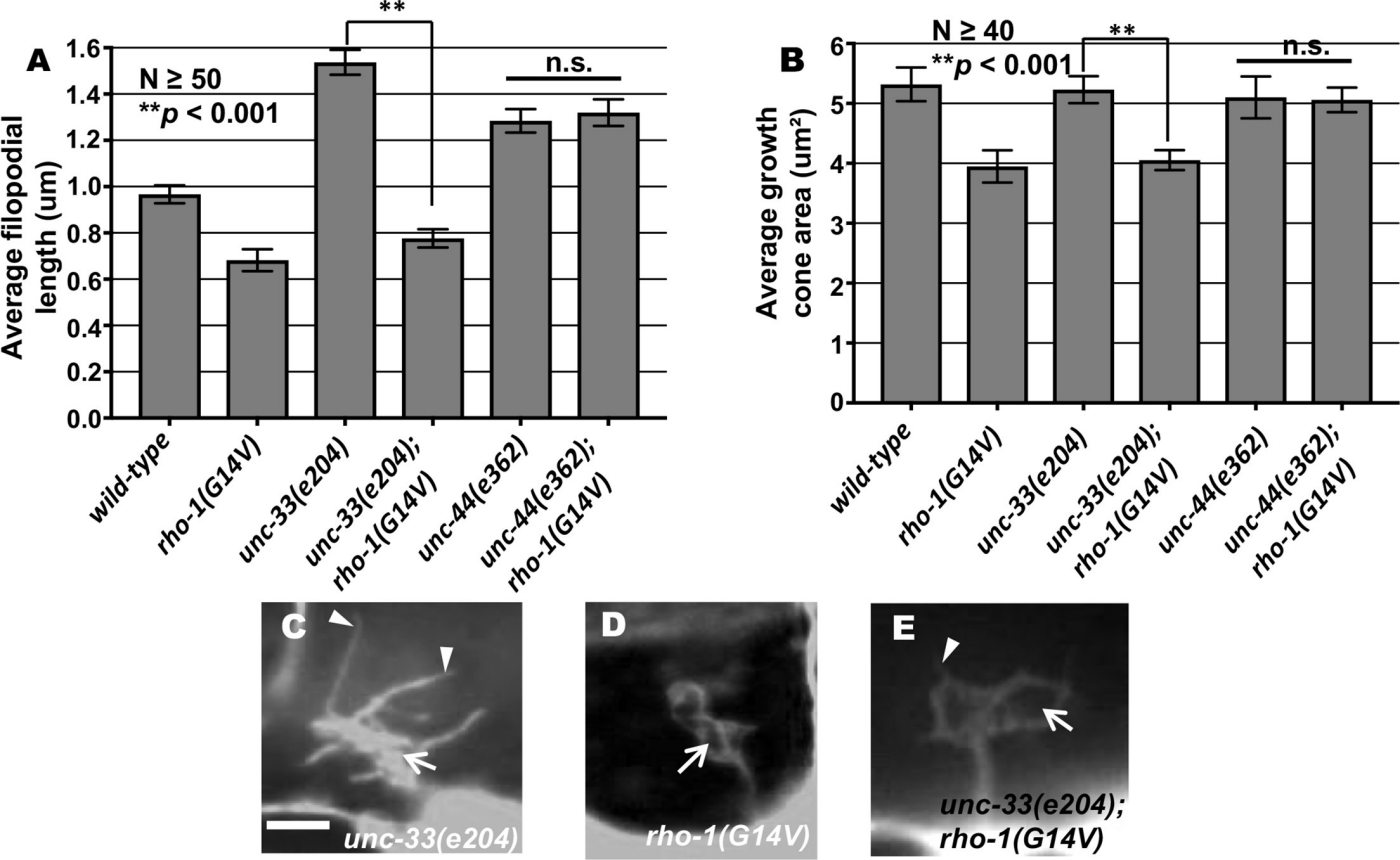
Genetic interaction *rho-1* with *unc-33* and *unc-44* in growth cone protrusion. (A-B) Quantification of VD growth cone filopodial length and growth cone area in single and double mutant animals as described in Fig 1. (A) Average filopodial length, in μm. (B) Growth cone area in μm^2^. Error bars represent 2x standard error of the mean; asterisks indicate the significant difference between the single mutant and the double mutant phenotype (**p < 0.001) determined by ANOVA. n.s., not significant. (C-E) Fluorescence micrographs of mutant VD growth cones as described in Fig 1. Arrows point to the growth cone and arrow heads indicate representative filopodia. Scale bar: 5μm.

*unc-5* loss of function results in unpolarized, overly-protrusive VD growth cones. Excess MT+ ends accumulate in *unc-5*, and dorsal polarity of F-actin accumulation and thus protrusion is lost [21, 22]. Activated *rho-1(G14V)* expression did not suppress the large growth cone area and long filopodial protrusions seen in *unc-5* mutants (i.e. double mutants resembled *unc-5* alone) (Fig 9). Furthermore, we observed no significant change in EBP-2::GFP and VAB-10ABD::GFP distribution in the VD growth cones as compared to *unc-5* mutants alone (Figs 10 and 11). This suggests that UNC-5 might have RHO-1-independent roles.

Double mutants of *unc-5* and dominant-negative *rho-1(T19N)* and *rho-1(RNAi)* showed significantly enhanced protrusion compared to single mutants, but did not exceed the additive effects of each (Fig 9F and 9G). This might reflect roles of these molecules that are independent of one another. Consistent with this notion, VD/DD lateral midline crossing axon guidance defects were significantly enhanced in *unc-5* double mutants with *rho-1(T19N)* and *rho-1(RNAi)* (Table 1).

### Activated RHO-1 suppresses *unc-33/CRMP* loss of function

The Collapsin-response mediator protein (CRMP) UNC-33 and the Ankyrin-like molecule UNC-44 are required for inhibition of growth cone protrusion of activated *myr*::*unc-40* and *myr*::*unc-5*. Loss of *unc-33* and *unc-44* results in VD growth cones resembling *unc-5* mutants, with increased protrusion, increased MT+ end accumulation, and loss of F-actin dorsal polarity [21, 23].

**Fig 12:**
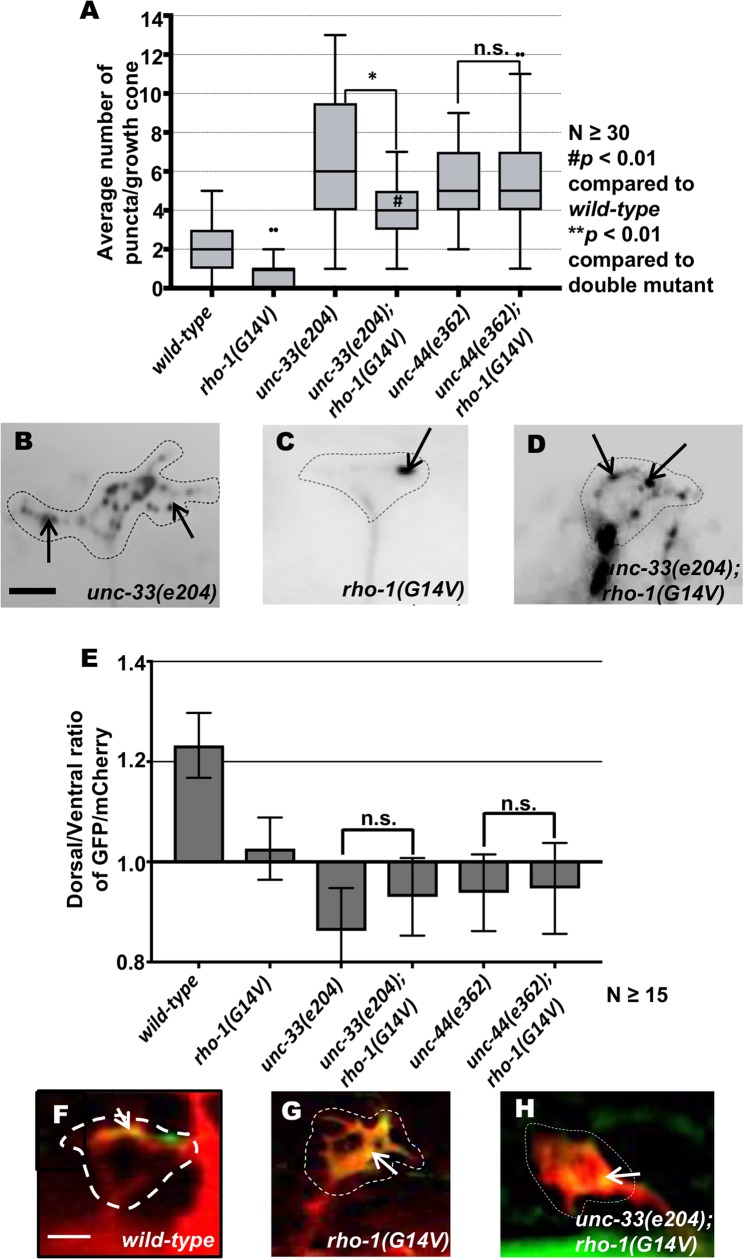
Genetic interactions of RHO-1 and *unc-33* and *unc-44* in EBP-2::GFP accumulation and F-actin polarity. (A) Quantification of the number of EBP-2::GFP puncta in wild-type and mutant animals as described in Fig 2E. Asterisks (*) indicate the significant difference between single mutants and the double mutant (*p < 0.01). Pound (#) indicates significant difference between wild-type and double mutant (*#p* < 0.01) determined by ANOVA. (B-D) Fluorescence micrographs of EBP-2 distribution in the VD growth cones. (B) An *unc-33(e204)* growth cone with increased *ebp-2* puncta. (D) A *rho-1(G14V)* small and inhibited growth cone with significantly fewer *ebp-2* puncta. (E) An *unc-33(e204); rho-1(G14V)* small and inhibited growth cone with increased *ebp-2* puncta. Arrows indicate representative EBP-2::GFP puncta. Dashed lines indicate the growth cone periphery. Dorsal is up and anterior is left. Scale bar: 5 μm. (E) The average dorsal-to-ventral ratio of GFP/mCherry from multiple growth cones in wild-type as described in Fig 2E. Error bars represent 2x standard error of the mean; n.s. indicates no significant difference between *unc-33* and *unc-44* single mutants and their respective double mutants determined by ANOVA. (F-H) Representative images of VD growth cones with cytoplasmic mCherry in red (a volumetric marker) and the VAB-10ABD::GFP in green. Areas of overlap are yellow (arrows). Scale bar: 5 μm. Dashed lines indicate the growth cone periphery. Dorsal is up and anterior is left.

Double mutants of *unc-33* and *rho-1(G14V)* resembled those of activated *rho-1(G14V)* mutants alone, with a significant decrease in growth cone area and filopodial protrusions (Fig 11). Despite reduced protrusion and smaller growth cone size, EBP-2::GFP puncta accumulation was increased in double mutants of *unc-33* and *rho-1(G14V)* (Fig 12). By contrast, double mutants of *unc-44* with *rho-1(G14V)* resembled *unc-44* mutants, with excessive growth cone filopodial as evidenced with increased filopodial length and growth cone area, as well as an increase in EBP-2 puncta distribution (Figs 11 and 12). Double mutants of *unc-33* and *unc-44* with *rho-1(G14V)* showed no significant change in F-actin distribution as compared to single mutants alone (Fig 12). These complex interactions reveal a differentiation of function between UNC-33/CRMP and UNC-44/Ankyrin in interaction with RHO-1 in growth cone morphology regulation.

## Discussion

Previous studies indicate that directed outgrowth of the VD growth cones away from UNC-6/Netrin involves a polarity/protrusion mechanism [21–23]. UNC-6/Netrin first polarizes protrusion to the dorsal side of the growth cone, and then regulates the extent of growth cone protrusion, with the receptor UNC-40 stimulating protrusion dorsally and the UNC-5 receptor inhibiting protrusion ventrally, resulting in directed dorsal growth away from UNC-6/Netrin. Growth cone polarity is reflected in F-actin polarity, with F-actin distribution biased to the dorsal side of the growth cone (i.e. the protrusive side). Growth cone protrusion correlates with the presence of MT+ ends, and MTs are pro-protrusive in the VD growth cones [21]. UNC-6/Netrin, its receptors UNC-5 and UNC-40, Rac GTPases, and UNC-33/CRMP all regulate both growth cone polarity and protrusion [21–23]. UNC-5 and UNC-33 normally inhibit growth cone protrusion in part by restricting MT+ end accumulation in growth cones [21].

Our results here show that the small GTPases RHO-1 and the Rho Guanine nucleotide Exchange factor RHGF-1 mediate inhibition of growth cone protrusion and are required to limit MT+ end accumulation in growth cones, similar to UNC-5 and UNC-33. However, RHO-1 and RHGF-1 had no effect on growth cone polarity (i.e. mutants did not affect dorsally-biased distribution of filopodial protrusion and F-actin). Thus, RHO-1 and RHGF-1 specifically affect VD growth cone protrusion, and not polarity. Activated RHO-1 was epistatic to *rhgf-1* loss of function (i.e. growth cones in double mutants displayed inhibited filopodial protrusions and a significant reduction in EBP-2 puncta distribution similar to activated *rho-1* alone), consistent with the known role of RHGF-1 as an upstream Rho activator.

Previously, missense mutations in *unc-40* and *unc-*6 uncoupled protrusive growth functions from polarity in neurons with axons that grow toward UNC-6 [24]. Our results demonstrate that polarity and protrusion can also be uncoupled in growth cones that grow away from UNC-6. Models of growth cone directed outgrowth along chemotactic gradients imply that growth cone polarity and protrusion are intimately linked, as differing concentrations of guidance cue are thought to differentially regulate protrusion across the growth cone, resulting in polarized growth. Our results show that polarity and protrusion can be independently regulated, consistent with previous results [24].

Genetic studies suggest a complex interaction of RHO-1 and RHGF-1 with UNC-5 and UNC-33. The data are consistent with the idea that RHO-1 and RHGF-1 act in the UNC-5 pathway as well as in a parallel pathway (RHGF-1 was required for the effects of activated MYR::UNC-5, and activated RHO-1 did not suppress *unc-5* loss of function). Additionally, activated RHO-1 suppressed the large, protrusive growth cones of *unc-33* loss-of-function, but did not decrease MT+ end accumulation in these small growth cones. This suggests that UNC-33 might act downstream of RHO-1 in MT accumulation, and that RHO-1 has an UNC-33-independent role in protrusion. While we do not fully understand the nature of these interactions at this point, our data clearly show that RHO-1 and RHGF-1 interact with UNC-6/Netrin signaling to regulate growth cone protrusion and MT organization during growth cone outgrowth.

### RHO-1 regulates growth cone protrusion and EBP-2 distribution

Expression of activated RHO-1(G14V) resulted in VD growth cones with a marked decrease in growth cone protrusion and EBP-2 puncta distribution (Figs 1 and 2). Expression of the dominant negative form of RHO-1(T19N) in the VD neurons and *rho-1(RNAi)* resulted in increased protrusion and EBP-2::GFP accumulation. MT+ ends in the growth cone periphery (Figs 1 and 2). Notably, neither dominant-negative RHO-1(T19N) or *rho-1(RNAi)* resulted in altered growth cone polarity and F-actin dorsal bias (Figs 1 and 2), suggesting that RHO-1 might specifically affect growth cone protrusion but not polarity.

Previous work has identified roles of the Rho GTPases in regulation of both microtubules and actin [46]. RhoA has been shown to regulate formation of contractile actin structures such as stress fibers and promote stabilization of microtubules [47, 48] through actomyosin contraction. In cultured growth cones, RhoA is involved in F-actin retrograde flow, wherein actin filaments in the periphery undergo constant retrograde transport to growth cone body [49–52]. RhoA activates RhoA kinase (ROCK), which activates contractility by phosphorylating the regulatory myosin light chain (MLC). This actin retrograde flow is thought to restrict MTs from the growth cone through physical association with these actin filaments undergoing retrograde flow, thereby reducing leading edge protrusion resulting in growth cone collapse and retraction [50, 53]. Growth cone advance can occur when this actin-MT linkage is disrupted or when actin becomes attached to the substrate (the “clutch” hypothesis) [54] resulting in anterograde flow over the anchored actin filaments. One hypothesis explaining our results is that, in VD growth cones, RHO-1-mediated retrograde flow of actin restricts MT+ ends from the growth cones, and when RHO-1 activity is reduced, more MTs enter the growth cones resulting in increased growth cone protrusion. RHO-1 does not control growth cone polarity. We envision that it controls the general entry of pro-protrusive factors into the growth cone, possibly delivered to the growth cone by microtubules. The disposition of these pro-protrusive factors then depends on earlier growth cone polarity. In other words, where these pro-protrusive factors are active, at the dorsal leading edge, depends on growth cone polarity. When more pro-protrusive factors are delivered as a result of *rho-1* loss, more protrusion occurs, but at the normal location.

### The Rho GEF RHGF-1 acts with RHO-1 to inhibit growth cone protrusion and MT accumulation

Loss of *rhgf-1* resulted in increased growth cone protrusion and accumulation of EBP-2::GFP, similar to but more pronounced than dominant-negative RHO-1(T19N) and *rho-1(RNAi)* (Figs 3 and 4). Furthermore, *rhgf-1* mutants had no effect on growth cone polarity of protrusion or F-actin distribution (Fig 4). RHGF-1 might be an activator of RHO-1 to inhibit growth cone protrusion and MT accumulation. Consistent with this idea, activated *rho-1* was epistatic to *rhgf-1* loss-of-function (i.e. activating RHO-1 bypasses the need for RHGF-1). Growth cones in these double mutants displayed inhibited protrusion and reduction in MT distribution similar to activated *rho-1* alone, suggesting that RHGF-1 acts as an upstream RHO-1 regulator in this process (Figs 5 and 6).

Previous studies in *Drosophila* S2 cells have shown that the RHGF-1 homolog, DRhoGEF2, induces contractile cell shape changes by regulating myosin II dynamics via Rho1 pathway. Furthermore, DRhoGEF2 associates with tips of growing MTs and travels to the cell cortex [45]. In *C*. *elegans*, RHGF-1 functions through Rho and ROCK to activate the MAPKKK DLK-1 during MT disruption, triggering synaptic branch retraction and overgrowth of PLM neurites ultimately leading to neuronal remodeling [37]. Possibly, RHGF-1 activates RHO-1 to mediate a potential retrograde flow of F-actin to restrict MT accumulation in the growth cone.

### RHGF-1 is required for the inhibitory effects of MYR::UNC-5 and MYR::UNC-40

*rhgf-1* loss-of-function suppressed the inhibitory effects of activated *myr*::*unc-40* and *myr*::*unc-5* on growth cones. Double mutant growth cones resembled those of *rhgf-1* alone, with increased protrusion and EBP-2::GFP puncta (Figs 7 and 8). That RHGF-1 is required for the effects of constitutively active MYR::UNC-40 and MYR::UNC-5 suggest that RHGF-1 acts downstream of MYR::UNC-5 and MYR::UNC-40. However, it is possible that RHGF-1 defines a parallel pathway. In any event, the inhibitory effects of MYR::UNC-5 and MYR::UNC-40 require functional RHGF-1.

### Activated RHO-1(G14V) cannot compensate for loss of UNC-5 in growth cone inhibition

Receptors to several attractive or repulsive guidance cues signal through complex pathways through the Rho family of small GTPases to direct changes in growth cone cytoskeletal organization [55, 56], and Rho activity is thought to be induced by “repulsive” cues [57]. Loss of the UNC-6/Netrin receptor *unc-5* has been shown to cause excessively large VD growth cones with increased protrusion and excess EBP-2::GFP accumulation [21, 22].

If RHO-1 is activated by UNC-5, we expect that activated *rho-1(G14V)* would be epistatic to *unc-5* loss-of-function. This was not the case, as growth cones of *rho-1(G14V); unc-5(lof)* double mutants resembled those of *unc-5(lof)* alone, with increased protrusiveness and EBP-2::GFP accumulation (Figs 9 and 10). Possibly, loss of UNC-5 affects multiple parallel pathways, including RHO-1, and activation of the RHO-1 pathway alone cannot compensate for loss of UNC-5. Alternately, RHO-1 might act in parallel to UNC-5. That RHGF-1 function is required for the effects of activated MYR::UNC-5 and MYR::UNC-40 suggests that RHGF-1 (and by extension RHO-1) might, in part, act in the UNC-5 pathway directly.

### UNC-33/CRMP is required for activated RHO-1(G14V) restriction of EBP-2::GFP

Previous studies have shown that the *C*. *elegans* UNC-33/CRMP is required in a pathway downstream with Rac GTPases for inhibition of growth cone protrusion in response to UNC-6/Netrin [23]. *unc-33* loss-of-function mutants show large protrusive growth cones with excess EBP-2 accumulation in the growth cones, similar to *unc-5*. While activated RHO-1(G14V) did not suppress the excessively-protrusive growth cones of *unc-5* mutants, it did suppress those of *unc-33* (Fig 11). Protrusion of growth cones of *rho-1(G14V); unc-33* double mutants resembled *rho-1(G14V)* alone (i.e. protrusion was reduced and growth cones were small).

Interestingly, despite their small size, inhibited *unc-33; rho-1(G14V)* growth cones displayed increased EBP-2 puncta compared to wild-type animals, but significantly lower than *unc-33* mutants alone (Fig 12). Thus, activated RHO-1(G14V) can fully suppress excess protrusion, but not EBP-2::GFP accumulation, of *unc-33* mutants. Together, these results suggest that UNC-33 is required for activated RHO-1(G14V) to restrict MTs from growth cones. They also suggest that RHO-1 has a role in protrusion that is independent of MT accumulation, as protrusion was reduced in *rho-1(G14V); unc-33* double mutants despite excess MT accumulation.

UNC-44/Ankyrin is required to properly localize UNC-33/CRMP to the axons [58], and mutants are phenotypically indistinguishable in the VD growth cones (both are required to polarize protrusion and F-actin and to inhibit protrusion and EBP-2::GFP accumulation) [21, 23]. However, *unc-44* loss was completely epistatic to activated RHO-1(G14V), including both protrusion and EBP-2::GFP accumulation. This suggests that UNC-44/Ankyrin has a role that is independent of UNC-33/CRMP involving non-MT-based regulation of protrusion. The FMO flavin monooxygenases inhibit growth cone protrusion with UNC-5 [30], possibly in an actin-based manner similar to MICAL [58, 59]. Possibly, UNC-44/Ankyrin acts in this pathway or another independently from UNC-33/CRMP.

### Summary

**Fig 13:**
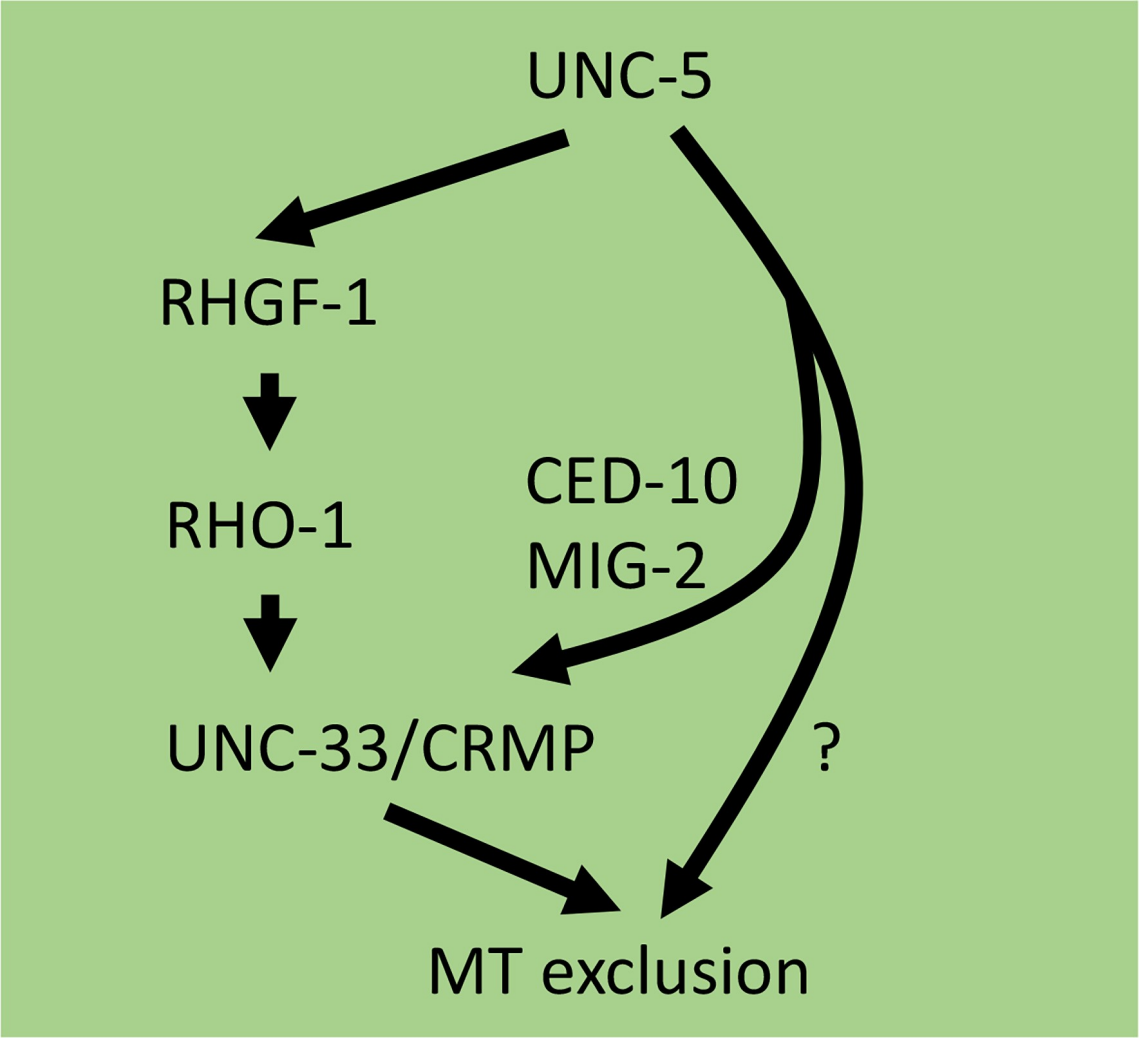
Possible interactions of RHO-1 in growth cone MT exclusion. UNC-5 might activate RHGF-1 and thus RHO-1, and UNC-33/CRMP is required for RHO-1 activity to exclude MTs from the growth cone. UNC-5 might activate Rac GTPases in parallel to drive MT exclusion via UNC-33/CRMP. UNC-5 might also engage a parallel pathway to drive MT exclusion.

Our results show that RHO-1 and the Rho activator GEF RHGF-1 are required to inhibit VD growth cone protrusion and to restrict EBP-2::GFP puncta accumulation in growth cones, possibly downstream of the UNC-6/Netrin receptor UNC-5. One potential scenario for how these molecules interact is shown in Fig 13. UNC-5 might activate RHGF-1 and thus RHO-1, and UNC-33/CRMP might then be required to exclude MTs from growth cones in response to RHO-1 activation. In parallel, the Rac GTPases CED-10 and MIG-2 also act with UNC-33/CRMP to regulate MT exclusion [21].

CRMP interactions with Rho, actin, and microtubules have been documented in other systems. In cultured mammalian neurons, CRMP interacts with F-actin and with tubulin dimers to promote microtubule assembly [60, 61], and expression of CRMP2 can alter Rho-GTPase-driven neurite morphology. Co-expression of Crmp-2 with activated Rho can promote cell spreading and neurite growth and this function of Crmp-2 is regulated by Rho Kinase [62]. Furthermore, CRMP-2 has been shown to be phosphorylated by Rho Kinase II [63, 64] which disrupts the association of mature full-length CRMP-2 with tubulin heterodimers so that tubulin cannot be transported to the plus ends of microtubules for assembly [61] causing neurite retraction and growth cone collapse [65]. This reduced binding capacity to tubulin by phosphorylated CRMP-2, can be reversed by inhibiting RhoA activity [66]. Thus, RHO-1 may regulate growth cone protrusion and MT distribution through the phosphorylation activity of UNC-33/CRMP possibly through the same pathway or in parallel to it.

If RHO-1 is indeed involved in F-actin retrograde flow, the role of UNC-33 might be to link F-actin to microtubules, such that in an *unc-33* mutant, MTs are not excluded despite retrograde flow (including in the activated RHO-1(G14V) background). RHO-1 might have an additional non-UNC-33 and non-MT-dependent role in inhibiting protrusion, along with UNC-44, possibly involving actin.

RHO-1 is a key negative regulator of growth cone protrusion and MT accumulation that acts specifically in the protrusion aspect of the polarity/protrusion model of directed growth cone migration away from UNC-6/Netrin. The separability of growth cone polarity and protrusion indicate that these are controlled by distinct mechanisms. Possibly, short-range interactions with UNC-6/Netrin result in growth cone polarity, and longer-range interactions (*e*.*g*. diffusible UNC-6/Netrin) maintain polarity and regulate protrusion as the growth cone moves away from the UNC-5/Netrin source. In the SOAL and polarity/protrusion a models, chemotactic gradients are not rerquired to explain directed outgrowth.

## Materials and methods

### Genetic methods

Experiments were performed at 20°C using standard *C*. *elegans* techniques [67]. Mutations used were LGIV: *unc-5(e53* and *e152)*, *unc-33(e204*), *unc-44(e362*); *lqIs128* [*Punc-25*::*myr*::*unc-40*] LGX: *rhgf-1(gk217*, *ok880 and gk292502)*, *lqIs170* [*rgef-1*::*vab-10ABD*::*gfp*]. Chromosomal locations not determined: *lqIs279* [*Punc-25*::*ebp-2*::*gfp*] by integration of *lqEx809*, *lhIs6* [*Punc-25*::*mCherry*], *lqIs296* [*Punc-25*::*myr*::*unc-5*], *lqIs312* [*Punc-25*::*rho-1(G14V)*] by integration of *lqEx1043*, *lqIs314* [*Punc-25*::*rho-1(T19N)*] by integration of *lqEx1070*. Extrachromosomal arrays were generated using standard gonadal injection [69] and include: *lqEx999* and *lqEx1000* [*Punc-25*::*myr*::*unc-40; Pgcy-32*::*yfp*], *lqEx1131*, *lqEx1132*, *lqEx1133* and *lqEx1134* [*Punc-25*::*rho-1 RNAi; Pgcy-32*::*yfp*], *OX347* [*Prgef-1*::*vab-10ABD*::*gfp; ttx-3*::*rfp*]. Multiple (≥3) extrachromosomal transgenic lines of *Punc-25*::*ebp-2*::*gfp*, *Punc-25*::*rho-1(G14V)* and *Punc-25*::*rho-1(T19N)* were analyzed with similar effect, and one was chosen for integration and further analysis.

### VD/DD axon guidance defects

In *wild-type*, and average of 16 of the 19 commissures of the VD/DD axons are distinguishable, as commissural axons sometimes run together as a bundle and cannot be resolved. For these experiments, 100 animals were scored for an average total of 1600 axons. In Table 1, “% defective VD/DD axon guidance” includes axon wandering greater than 45 degrees laterally, axon branching, and premature axon termination. As axon guidance defects are nearly completely penetrant in *unc-5* mutants, another guidance metric was used. “% failure to cross lateral midline” were axons that failed to extend dorsally past the lateral midline. Significance of difference was determined by Fisher’s Exact Test.

### Growth cone imaging

VD growth cones were imaged and quantified as previously described [22]. Briefly, animals at ~16 h post-hatching at 20°C were placed on a 2% agarose pad and paralyzed with 5mM sodium azide in M9 buffer, which was allowed to evaporate for 4 min before placing a coverslip over the sample. Some genotypes were slower to develop than others, so the 16 h time point was adjusted for each genotype. Growth cones were imaged with a Qimaging Rolera mGi camera on a Leica DM5500 microscope. Images were analyzed in ImageJ, and statistical analyses done with Graphpad Prism software. As described in [22, 23], growth cone area was determined by tracing the perimeter of the growth cone body, not including filopodia. Average filopodial length was determined using a line tool to trace the length of the filopodium. Unless otherwise indicated, ≥25 growth cones were analyzed for each genotype. These data were gathered in ImageJ and entered into Graphpad Prism for analysis. Analysis of Variance (ANOVA) was used to determine significance of difference between genotypes. Any of the VD growth cones visible at the time of imaging were scored (VD2-VD13), and we did not focus on any single VD growth cone for analysis.

### VAB-10ABD::GFP imaging

The F-actin binding domain of VAB-10/spectraplakin fused to GFP has been used to monitor F-actin in *C*. *elegans* [68, 69]. We used it to image F-actin in the VD growth cones as previously described [22]. To control for variability in growth cone size and shape, and as a reference for asymmetric localization of VAB-10ABD::GFP, a soluble mCherry volume marker was included in the strain. Growth cones images were captured as described above. ImageJ was used image analysis to determine asymmetric VAB-10ABD::GFP localization. For each growth cone, five line scans were made from dorsal to ventral. For each line, pixel intensity was plotted as a function of distance from the dorsal leading edge of the growth cone. The average intensity (arbitrary units) and standard error for each growth cone was determined. For dorsal versus ventral comparisons, the pixel intensities for VAB-10ABD::GFP were normalized to the volumetric mCherry fluorescence in line scans from the dorsal half and the ventral half of each growth cone. This normalized ratio was determined for multiple growth cones, and the average and standard error for multiple growth cones was determined. Statistical comparisons between genotypes were done using ANOVA on these average normalized ratios of multiple growth cones of each genotype.

### EBP-2::GFP imaging

EBP-2::GFP has previously been used to monitor microtubule plus ends in other *C*. *elegans* cells including neurons [70–72]. We constructed a transgene consisting of the *unc-25* promoter driving expression of *ebp-2*::*gfp* in the VD/DD neurons. In growth cones, a faint fluorescence was observed throughout the growth cone, resembling a soluble GFP and allowing for the growth cone perimeter to be defined. In addition to this faint, uniform fluorescence, brighter puncta of EBP-2::GFP were observed that resembled the EBP-1::GFP puncta described in other cells and neurons. For each growth cone, the perimeter and filopodia were defined, and the EBP-2::GFP puncta in the growth cone were counted. For each genotype, the puncta number for many growth cones (≥25 unless otherwise noted) was determined. Puncta number displayed high variability within and between genotypes, so box-and-whiskers plots (Graphpad Prism) were used to accurately depict this variation. The grey boxes represent the upper and lower quartiles of the data set, and the “whiskers” represent the high and low values. Dots represent major outliers. Significance of difference was determined by ANOVA.

### Transgenic RNA-mediated gene interference (RNAi)

We used a cell-specific transgenic RNAi approach as described previously [40]. Fragments of the *rho-1* coding region was amplified by PCR and inserted behind the *unc-25* promoter in a plasmid (primer and plasmid sequences available upon request). A “sense” and “antisense” orientation relative to the *unc-25* promoter was isolated. An equimolar mixture of the sense and antisense plasmids was used to construct transgenic animals. These transgenic animals were predicted to express both sense and antisense RNAs driven by the *unc-25* promoter in the VD/DD motor neurons, which was expected to trigger a double-stranded RNA response in these cells (RNAi).
